# Measurement of the helicity asymmetry *E* for the reaction $\gamma p\rightarrow \pi ^0 p$: The CBELSA/TAPS Collaboration

**DOI:** 10.1140/epja/s10050-020-00334-2

**Published:** 2021-01-28

**Authors:** M. Gottschall, F. Afzal, A. V. Anisovich, D. Bayadilov, R. Beck, M. Bichow, K. Th. Brinkmann, V. Crede, M. Dieterle, F. Dietz, H. Dutz, H. Eberhardt, D. Elsner, R. Ewald, K. Fornet-Ponse, St. Friedrich, F. Frommberger, A. Gridnev, M. Grüner, E. Gutz, Ch. Hammann, J. Hannappel, J. Hartmann, W. Hillert, Ph. Hoffmeister, Ch. Honisch, T. Jude, S. Kammer, H. Kalinowsky, I. Keshelashvili, P. Klassen, F. Klein, E. Klempt, K. Koop, B. Krusche, M. Kube, M. Lang, I. Lopatin, P. Mahlberg, K. Makonyi, V. Metag, W. Meyer, J. Müller, J. Müllers, M. Nanova, V. Nikonov, R. Novotny, D. Piontek, G. Reicherz, T. Rostomyan, A. Sarantsev, Ch. Schmidt, H. Schmieden, T. Seifen, V. Sokhoyan, K. Spieker, A. Thiel, U. Thoma, M. Urban, H. van Pee, D. Walther, Ch. Wendel, D. Werthmüller, U. Wiedner, A. Wilson, A. Winnebeck, L. Witthauer, Y. Wunderlich

**Affiliations:** 1 Helmholtz–Institut für Strahlen– und Kernphysik, Universität Bonn, Bonn, Germany; 2 Physikalisches Institut, Universität Bonn, Bonn, Germany; 3 Petersburg Nuclear Physics Institute, Gatchina, Russia; 4 Physikalisches Institut, Universität Gießen, Gießen, Germany; 5 Physikalisches Institut, Universität Basel, Basel, Switzerland; 6 Institut für Experimentalphysik I, Ruhr–Universität Bochum, Bochum, Germany; 7 Department of Physics, Florida State University, Tallahassee, FL 32306 USA

## Abstract

A measurement of the double-polarization observable *E* for the reaction $\gamma p\rightarrow \pi ^0 p$ is reported. The data were taken with the CBELSA/TAPS experiment at the ELSA facility in Bonn using the Bonn frozen-spin butanol (C$_4$H$_9$OH) target, which provided longitudinally-polarized protons. Circularly-polarized photons were produced via bremsstrahlung of longitudinally-polarized electrons. The data cover the photon energy range from $E_\gamma =600$ to 2310 MeV and nearly the complete angular range. The results are compared to and have been included in recent partial wave analyses.

## Introduction

Nucleons, protons and neutrons, are not elementary particles. They contain three light valence quarks. As a three-body system, the nucleon is expected to exhibit a large number of excitation modes (see e.g. [1–3]), and the prediction of hybrid baryons increases the number of expected resonances even further [4, 5]. Lattice QCD seems to confirm the large number of predicted states, at least when a quark mass corresponding to $m_\pi =396$ MeV is used [6]. In the mass range up to 1.7 GeV, the abundance of the predicted states as well as most of their masses correspond reasonable well to experiment. This is no longer true at higher masses. Here, many more resonances have been predicted than have been observed so far. To understand the problem of the *missing resonances* and the spectrum of baryons in general, different approaches have been suggested: possibly, (1) the three-quark dynamics is frozen to a quark-diquark system [7], (2) quark models might use the wrong degrees of freedom and the excited baryons could rather be generated dynamically by the interaction of mesons and octet or decuplet (ground-state) baryons [8–13] leading to a different spectrum of observed states, or (3) the number of excited baryons might be reduced in models based on AdS/QCD [14–16]. However, (1) predicts fewer resonances than observed, (2) and (3) do not give a prediction which resonances should be observed and which ones not. Recent surveys of the field can be found in [17–20].

Alternatively, the number of baryon resonances predicted in quark models could be correct, but a sizable fraction of them may have escaped discovery in appropriate experiments. The traditional path to the excitation spectrum of nucleons was the study of $\pi N$ scattering. For a long time, our knowledge on the excitation spectrum was based on the two classical analyses at Karlsruhe-Helsinki (KH) [21] and Carnegie Mellon (CM) [22], and the more recent analysis at George Washington University (GW) [23] which included high-precision data from meson factories and measurements of the phase-sensitive polarization state of the recoiling nucleon. Only recently, the experimental efforts at ELSA, GRAAL, JLab, MAMI, and Spring-8 have provided high-statistics data on photoproduction of mesons off nucleons, and several new nucleon resonances were discovered (see e.g. [24]). But still, the number of predicted states exceeds the number of observed states. There is hope that further data and further analyses will finally clarify the *missing resonance* problem.

Polarization experiments are key to improving the database of photoproduction reactions. For the two best studied photoproduction processes, $\gamma p\rightarrow \pi ^0 p$ and $\gamma p\rightarrow \pi ^+ n$, data were accumulated for several observables. Here, we quote a few recent measurements; references to data reported before the year 2000 can be found elsewhere [30]. The recent data cover the differential cross section $d\sigma /d\varOmega$ [31–37], the beam asymmetry $\varSigma$ [33, 38–40], the target asymmetry *T* [41–43], and the recoil polarization *P* [41, 42, 44, 45]. The single-polarization observables $\varSigma , T$ and *P* require the use of linearly-polarized photons, of transversely-polarized protons, or the analysis of the polarization of the outgoing protons, respectively. The observable *P* can, however, also be determined from a measurement using linearly-polarized photons and transversely-polarized protons [41, 42].

More information can be gained when two of the three polarizations are controlled experimentally. Photons can be polarized linearly or circularly, and target protons can be polarized along the photon-beam axis or in a transverse direction to measure the quantities *G* [46–48], *E* [49–53], *H* [41, 42], and *F* [43]. The correlation between the photon polarization and the recoil polarization yields the observables $O_{x^\prime },O_{z^\prime }, C_{x^\prime }, C_{z^\prime }$. The correlation between the target polarization and the recoil polarization is governed by $T_{x^\prime },T_{z^\prime }$, $L_{x^\prime }, L_{z^\prime }$. For $C_{x^\prime }$ and $C_{z^\prime }$, data have been published for a few specific energies and angles [44, 45, 54]. This is also the case for $O_{x^\prime }$ and $O_{z^\prime }$, where only a few older data points exist [55, 56]. To our knowledge, no direct measurements of the target-recoil polarization for single-$\pi ^0$ photoproduction have been undertaken. Following theory, not all 16 observables need to be determined to obtain a unique solution for the photoproduction amplitude. The scattering process is governed by four complex amplitudes, the so-called Chew-Goldberger-Low-Nambu (CGLN) amplitudes [57], and a minimum of seven carefully chosen measurements should be sufficient for their determination (up to an unknown phase). Due to the nonlinear relation between amplitudes and observables, an eighth measurement is required to resolve discrete ambiguities [58–60], and all measurements have to be precise and cover the full solid angle [61]. This approach leads—if successful—to the reconstruction of the four complex amplitudes for every bin in photon energy and angle. For every kinematic bin, one phase is arbitrary. The CGLN amplitudes can then be expanded into a series of electric and magnetic multipoles which drive the excitation of particular resonances. In practice, this expansion needs to be truncated using a finite number of multipoles only.

A more direct approach constructs the multipoles directly by fitting the data. In this approach, the minimum number of known observables can even be smaller than eight, depending on the number of contributing multipoles and the data quality (truncated partial wave analysis). At low energies, even a small number of observables can be sufficient to arrive at a unique solution [62, 63]. Below the 2$\pi$ threshold, only *S* and *P* waves contribute to $\pi ^0$ photoproduction and the strong-interaction phase is fixed due to the Watson theorem [64]. A measurement of differential cross sections $d\sigma /d\varOmega$ and the photon beam asymmetry $\varSigma$ is thus sufficient to determine the contributing multipoles in the first resonance region where the $\varDelta (1232)$ resonance dominates [65, 66]. With the number of known observables increasing, *S*, *P*, and *D* waves can be determined in an energy-independent fit to the data covering the second resonance region [41]. Here, higher partial waves, including resonance but also background contributions e.g. due to t-channel exchange, have been fixed to their contributions determined from an energy-dependent fit.

**Fig. 1:**
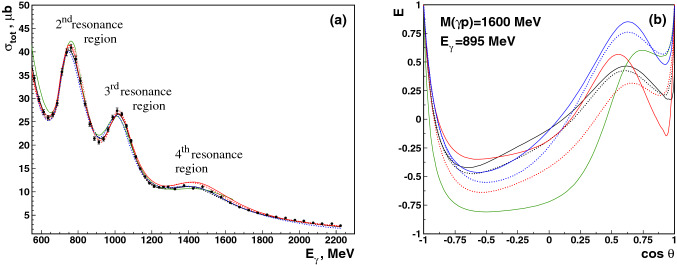
Left: Total $\gamma p \rightarrow p\pi ^0$-cross section. Right: Predictions for the double-polarization variable *E*. The different PWA solutions for which the predictions are shown, did not yet include the new polarization data discussed in this paper: BnGa 2011-02 (black solid) [24], BnGa 2011-01 (black dotted) [24], SAID CM12 (red solid) [25], SAID SN11 (red dotted) [28], JüBo 2015-B (blue solid) [26], JüBo 2013-01 (blue dotted) [27], MAID 2007 (green solid) [29]

The higher the incident photon energy, the more resonances contribute. This in turn makes the measurement of additional polarization observables necessary to finally pin down the contributing resonances. Figure 1 (left) shows fits to the $\gamma p\rightarrow \pi ^0 p$ total cross section. The data reveal two clear peaks and a smaller enhancement which are assigned to the 2$^\mathrm{nd}$, 3$^\mathrm{rd}$, and 4$^\mathrm{th}$ resonance regions where several resonances contribute with masses around 1500 MeV, 1650 MeV and 1900 MeV, respectively. The MAID, SAID, JüBo and BnGa partial wave analyses all reproduce the data well. However, even at rather low energies, the predictions for the double-polarization observable *E* spread over a wide range, see Fig. 1 (right). Obviously, the amplitudes included in the different PWA solutions shown in the figure are not identical (see also [67]). This is especially true if results from different PWA-groups are compared. This finally also leads to different resonances and resonance properties extracted based on the different PWAs.

In this paper, we report on a measurement of the double polarization observable *E* for the reaction $\gamma p\rightarrow \pi ^0p$ in the photon energy range from $E_\gamma =600$ MeV to $E_\gamma =2310$ MeV. Selected data have been presented in a Letter [51]. Here, we give experimental details and report the full data set on *E*.

The paper is organized as follows: This Introduction is followed by Sect. 2 which defines the double-polarization variable *E* and describes the experimental method. Section 3 introduces the experimental set up. Decisive ingredients for the polarization measurements presented here are of course the beam and target polarization, which are reviewed in Sect. 4. It is followed by Sect. 5 on the calibration procedures and the selection of the events. In Sect. 6, the analysis method used to determine the helicity asymmetry *E* is explained and the results on *E* are presented and discussed. The paper ends with a short summary.

## The double-polarization observable *E* for the reaction $\gamma p \rightarrow \pi ^0 p$

The double-polarization observable *E* is defined as the normalized difference of the cross sections for antiparallel and aligned spins of beam photons and target protons, $\sigma _{1/2}$ and $\sigma _{3/2}$, respectively:

1 $$\begin{aligned} E=\frac{\sigma _{1/2}-\sigma _{3/2}}{\sigma _{1/2}+\sigma _{3/2}}=\frac{\sigma _{1/2}-\sigma _{3/2}}{2\sigma _0}\,, \end{aligned}$$

where the unpolarized cross section $\sigma _0$ is written as $2\sigma _0=\sigma _{1/2}+\sigma _{3/2}$.

To measure the observable *E*, a circularly-polarized photon beam and a longitudinally-polarized target were needed. Circularly-polarized photons were produced using longitudinally-polarized electrons (see Sect. 4.1) which then transferred part of their polarization to the bremsstrahlung photon. Their polarization was monitored by Møller polarimetry [68]. Polarized protons were obtained by using the Bonn frozen-spin polarized butanol (C$_4$H$_9$OH) target (see Sect. 4.2).

The observable *E* can be expressed in terms of count rates in the following way:

2 $$\begin{aligned} E= \frac{N_{1/2}-N_{3/2}}{N_{1/2}+N_{3/2}}\frac{1}{P_TP_\odot }\frac{1}{d}\,. \end{aligned}$$

$N_{3/2}$ and $N_{1/2}$ are the count rates for the beam-target setting with the spins of the photon and proton parallel and antiparallel, $P_T$ is the target polarization, $P_\odot$ the beam polarization and *d* the dilution factor. The dilution factor takes into account that butanol contains not only polarizable protons in hydrogen, but also nucleons bound in carbon and oxygen nuclei, which are not polarizable. Thus, the fraction of free polarizable protons contributing to the reaction had to be determined and is given by the dilution factor *d*.

**Fig. 2:**
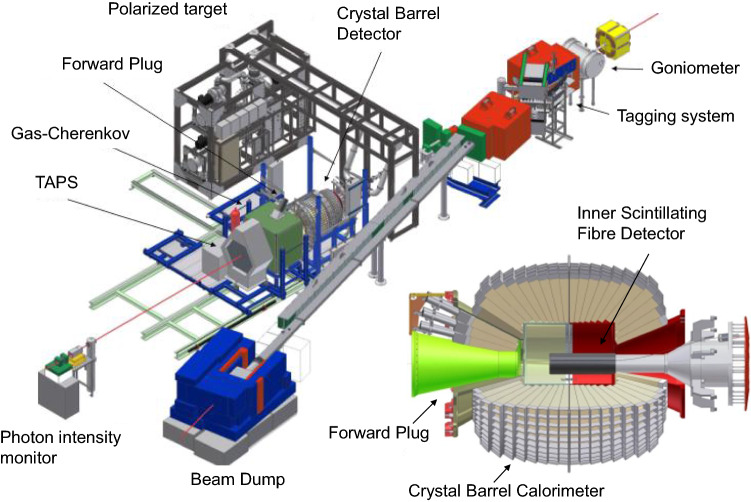
Experimental setup of the CBELSA/TAPS Experiment: full setup (left), more detailed view of the Crystal Barrel calorimeter with inner scintillating fibre detector and forward plug (lower right)

The measurement of *E* for $\gamma p \rightarrow \pi ^0 p$ required the identification of the reaction

3 $$\begin{aligned} \gamma p \rightarrow \pi ^0 p \rightarrow \gamma \gamma p \end{aligned}$$

and a separate measurement of the number of events $N_{3/2}$ and $N_{1/2}$. In addition, the dilution factor *d*, the target polarization $P_T$, and the beam polarization $P_\odot$ were needed.

After a calibration procedure (see Sects. 5.1–5.3) for the detector components, the energy of the bremsstrahlung beam-photon was deduced from a measurement of the energy loss of the corresponding electron (see Sect. 5.4). The four-vectors of photons produced in the reaction were reconstructed from the Crystal Barrel and TAPS electromagnetic calorimeters (see Sect. 5.5). In the 2$\gamma$ invariant mass spectrum, the $\pi ^0$ was clearly observed. The proton was identified in the forward direction by plastic scintillators in front of the crystals ($<30^\circ$) or at larger angles by a scintillating fiber detector surrounding the target (see Sect. 5.6). Its direction was determined either from its respective signal in one of the calorimeters or from the signal of the scintillating fiber detector. The kinematics of the event was then over-constrained by energy and momentum conservation, and the events of interest were identified nearly background free (see Sect. 5.7).

## Experimental setup

### Overview

An overview of the CBELSA/TAPS experiment is shown in Fig. 2. The experiment was located at the electron accelerator ELSA [69] in Bonn, Germany. ELSA provided either unpolarized or longitudinally-polarized electrons with energies up to 3.2 GeV (see Sect. 4.1). The electrons hit a radiator target inside a goniometer, where photons were produced via bremsstrahlung. For the data presented here, a Møller target was used as the bremsstrahlung target which allowed the monitoring of the electron polarization via Møller scattering in parallel to the data taking. The Møller target was a 20 $\mu$m thin foil with a magnetization of 8.2%, enclosing an angle of ±20$^\circ$ with the electron beam (see Sect. 4.1). After the bremsstrahlung process, the electron was deflected in a magnetic field and momentum-analyzed in a ladder of organic scintillator bars and fibers (see Sect. 3.2). The photon energy was then deduced from the energy difference of the incoming and the deflected electron after the bremsstrahlung process.

The bremsstrahlung photons impinged on the polarized target (see Sect. 4.2). The target was surrounded by a three-layer scintillation fiber detector used for the identification of charged particles and by the Crystal Barrel electromagnetic calorimeter consisting of 1230 CsI(Tl)-crystals. In the forward direction, below polar angles of 30$^\circ$, two further calorimeters, the Forward Plug (90 CsI(Tl) crystals) and the forward TAPS-wall (216 BaF$_2$ crystals), provided calorimetric information. Plastic scintillators in front of these crystals allowed for the identification of charged particles.

The signals from the forward calorimeters, the fiber detector and the tagging system were used in the first-level trigger. A second-level trigger identified the number of clusters in the Crystal Barrel calorimeter. In between the Crystal Barrel and the forward TAPS detector, a CO$_2$ Cherenkov counter vetoed signals of electron or positron hits due to the electromagnetic background produced in the target.

### The Tagging System

The Tagging System [70] consisted of 96 partly-overlapping scintillation bars with a width between 1.4 and $5\,$cm. These allowed us to tag electrons in the energy range between 2.1 and 82.5% of the electron beam energy $E_{0}$. To improve the energy resolution, the scintillation bars were complemented by 480 cylindrical scintillating fibers with 0.2 cm in diameter covering the electron energy range from 16.6 to 88.1% of $E_{0}$. Combined, this resulted in a tagging range for photons ($E_{\gamma }=E_0-E_e$, with $E_e$ the measured electron energy) from 11.9 to 97.9% with an energy resolution varying from $\sim$0.5 % $E_{0}$ at low photon energies to 0.1 % $E_{0}$ at high photon energies. While the high photon energy regime was only covered by bars, the very low photon energy regime was only covered by fibers. The bars and the fibers were read out by photomultipliers reaching a time resolution for the bars (fibers) of about 0.5 ns (1.2 ns) FWHM. The signals from the tagger bars were included in the first-level trigger of the experiment.

### Collimator and the beam dump

After the goniometer in the photon beamline, six collimators each with an inner (outer) diameter of 4 (20) mm and a length of 4 cm reduced the beam halo. Behind the collimators, a permanent magnet deflected low energetic charged particles coming along the beamline, which then hit a lead wall. Electrons from the primary beam not producing bremsstrahlung were deflected into a beam dump. The latter consisted of about 70 t of steel and was placed far behind the calorimeters to avoid background in the detector systems coming from the beam dump.

### Targets

The main data used for this work were taken with the Bonn frozen spin target. The butanol (C$_4$H$_9$OH) target material was placed inside a target container of 2 cm in length and 2 cm in diameter, which was immersed in liquid He (for details on the target polarization, see Sect. 4.2). In a butanol target, polarizable free protons as well as nucleons bound in the unpolarized carbon and oxygen nuclei contribute to the count rate. To determine the contribution from the bound nucleons, a carbon foam target was used inside the cryostat of the polarized target. The target nose was also filled with liquid helium to keep the conditions comparable to the butanol measurements. The foam target had approximately the same density as the carbon and oxygen component of the butanol target. For further tests data were were taken with a liquid hydrogen (LH2) target—as used, e.g., in [71] and [72].

### The Inner Scintillating Fiber Detector

The target was surrounded by the Inner Scintillating Fiber Detector [73]. It was 400 mm long, had an inner (outer) diameter of 116 mm (131 mm), and covered the polar angle range of $21^\circ< \theta < 167^\circ$. The detector consisted of 513 scintillating fibers with a diameter of 2 mm in 3 layers. The outer layer (191 fibers) was positioned parallel to the beam axis, the middle layer (165 fibers) was oriented at an angle of $+25.7^\circ$, and the innermost (157 fibers) at an angle of $-24.5^\circ$ with respect to the beam axis. The angles resulted from the requirement for the bent fibers to go exactly halfway around the detector. Charged particles were identified and their impact points reconstructed by coincident hits in two or three layers. The readout was organized via 16-channel photomultipliers connected to the fibers via light guides. The inner detector was included in the first-level trigger. A valid inner detector trigger signal was defined by two out of three layers firing.

### The calorimeters

The electromagnetic calorimeters of the detector system covered the full azimuthal range and polar angles from $1^{\circ }$ in the forward direction to $156^{\circ }$ in the backward direction. The combined solid-angle coverage was about 95 % of 4$\pi$.

**The Crystal Barrel calorimeter and the Forward Plug ** The Crystal Barrel calorimeter consisted of 1230 CsI(Tl) crystals, each crystal pointing to the target center. Its 21 rings were arranged in a barrel shape around the production target, 10 rings covered the forward hemisphere, 11 rings the backward hemisphere. Every ring consisted of 60 crystals, each covering an azimuthal- and polar-angle range of $6^{\circ }$ except for the last ring in the backward direction which consisted of 30 crystals only, each covering an azimuthal-angle range of $12^{\circ }$. The crystals were read out via a photo-diode mounted at the edge of a wavelength shifter collecting the light emitted by the crystal at its backside [74]. This component of the calorimeter covered the polar angles of 30$^{\circ }$–156$^{\circ }$.

A fast cluster encoder (FACE) [75] based on a cellular logic defined the number of contiguous clusters of crystal hits with energies above $\sim$15 MeV, typically within less than 10 $\mu$s. This information was used as a second-level trigger. For rejected events, a fast reset was generated which cleared the readout electronics in less than 15 $\mu$s.

In the forward direction, the Crystal Barrel calorimeter was complemented by the Forward Plug (FP), which consisted of additional 90 CsI(Tl) crystals arranged in three rings covering the polar angle from $11.2^{\circ }$ to $27.5^{\circ }$. Due to an additional holding structure, there was an area between the two detectors, where the acceptance was slightly reduced. The Forward Plug was read out by photomultipliers providing a fast enough timing signal to be used in the first-level trigger. A cluster finder provided the number of clusters in the FP [76]. It was based on a comparison of the crystal hit pattern in the FP with a Field Programmable Gate Array (FPGA) internal lookup table and delivered the number of clusters in less than 200 ns. It could thus be incorporated in the first level-trigger. Typically the cluster finder considered crystals with more than 25 MeV as hit crystals. For the identification of charged particles, 180 overlapping scintillator plates were mounted in two layers in front of these crystals. The azimuthal granularity of each scintillator plate in angle was $12^\circ$. Each scintillator in the first layer, close to the crystals, covered one of the 90 CsI(Tl) crystals. The second layer was turned by $6^\circ$ relative to the first one, covering now one half of two crystals. This doubled the $\phi$ angular resolution for the charged particles.

The Crystal Barrel calorimeter (including the Forward Plug) was optimized for photon detection. The energy resolution was empirically determined to be [74]

4 $$\begin{aligned} \frac{\sigma _E}{E}\;\approx \;\frac{2.4\,\%}{~({E~[\mathrm{GeV}]})^{1/4}~}\,. \end{aligned}$$

Since photons produce a shower in the calorimeter, an angular resolution better than the crystal granularity can be reached. As an example, using photons with energies above 200 MeV (500 MeV) which were not hitting the detector boundaries, an angular resolution of better than $1.75^{\circ }$ ($1.4^{\circ }$) was reached by determination of the shower center. The angular resolution improved for higher-energy photons. The given resolution takes into account the extension of the butanol target. For a point-like target, the respective resolutions would improve to $1.5^{\circ }$ ($1.1^{\circ }$) [77].

**The TAPS detector ** At a distance of 2.10 m behind the target center, the TAPS detector with 216 BaF$_2$ crystals [78] closed the hole in the forward direction. It had a high granularity and covered polar angles from $12^{\circ }$ down to $1^{\circ }$ . This calorimeter reached an energy resolution [79] of

5 $$\begin{aligned} \frac{\sigma _E}{E}\;=\;\frac{0.59\,\%}{\sqrt{E~[\mathrm{GeV}]}}+1.9\,\%\, \end{aligned}$$

for photons, and a time resolution of $\sigma \approx 370$ ps measured relative to the tagging system. The 5-mm-thick scintillator plates in front of the crystals discriminated charged against uncharged particles. The fast photomultiplier readout of the TAPS BaF$_2$-crystals allowed the inclusion of its signals into the first-level trigger. TAPS was divided into four trigger sectors (each 25 % of TAPS) and provided two types of first-level trigger signals. The TAPS1-trigger was provided by at least one trigger sector showing a trigger signal above 80 MeV. The TAPS2-trigger indicated that at least two trigger sectors had fired with an energy deposit above 80 MeV; in this case, signals in the two innermost rings were excluded since they suffered from a large $e^+e^-$ background. In the case of the TAPS2 trigger, no further hits in the other calorimeters were required, in contrast to the TAPS1 trigger.

### The Cherenkov detector

A CO$_2$ gas Cherenkov detector was used to identify electromagnetic background. It consisted of a parabolic mirror, which focussed the Cherenkov light onto a single photomultiplier. With an energy threshold of $E_\mathrm{thres}=17.4$ MeV for electrons and $E_\mathrm{thres}=4.76$ GeV for charged pions, it was well suited to suppress pair production and Compton events at the trigger level without affecting charged hadrons.

### The gamma intensity monitor and flux monitor

At the end of the beamline, directly in front of the photon beam dump, the gamma intensity monitor (GIM) consisted of 16 PbF$_2$ crystals. Photons impinging on the GIM induced an electromagnetic shower whose Cherenkov light signals were read out by photomultipliers. For photon rates $\gg$ 1 MHz, the GIM efficiency decreased due to deadtime effects. Therefore, a second detector was placed in front of the GIM. This flux monitor consisted of a 100-$\mu$m-thick Pb foil used as a thin conversion target followed by two organic scintillators to detect the $e^+e^-$-pairs. A third scintillator placed in front of the Pb foil was used for the suppression of charged-particle background. Calibrated relative to the GIM at low photon rates, the flux monitor counted only a well known fraction of the total photon flux. It was also used to determine the rate-dependent GIM detection efficiency.

### The trigger

Trigger conditions were imposed at two levels. The first-level trigger required a valid hit in the tagger not vetoed by the Cherenkov counter. This condition was combined with specific first- and second-level configurations for at least two detected particles in the calorimeters to enhance the fraction of useful data on disk. At least two hits (above the respective trigger threshold) in any combination of the Forward Detector and TAPS hits triggered the readout. If there was only one hit in these two detector components (or no hit, combined with a hit in the inner detector), the second-level trigger FACE had to identify at least one cluster (or at least two clusters) in the Crystal Barrel Detector. These trigger conditions covered all possible combinations for having at least two clusters somewhere in the calorimeters.

## The polarized beam and target

###  Photon polarization

**The source of polarized electrons: ** Polarized electrons were produced via photoemission from a GaAs/GaAsP strained-layer superlattice photocathode. By optical pumping using circularly-polarized laser light, electrons in a specific spin state were transferred from the valence to the conduction band. Due to the energy gap between the conduction band and the vacuum (electron affinity (EA) of typically 5.2 eV), these electrons could not escape to the vacuum without further measures. In order to lower the EA, a 5-nm-thick surface layer of the crystal was heavily p-doped ($5 \times 10^{19}/\mathrm{cm}^3$). A further reduction of the EA to negative values was achieved by depositing a monolayer of cesium and oxygen. This Cs-O layer caused the vacuum level to fall below the conduction band, and the electrons could tunnel through the remaining thin potential barrier. Using a flashlamp-pumped Ti:Sa laser, microsecond-long electron pulses with a polarization above 80 % and a pulse current of 120 mA were generated. The electrons underwent a first acceleration in the source and were transferred first to a linear accelerator (Linac), guided through magnetic deflection and focusing fields. An additional electrostatic deflector turned the electron polarization into transverse direction. The electrons were pre-accelerated in the Linac and the following Booster Synchrotron, injected into the ELSA ring, post-accelerated to their final energy, and then slowly extracted for the experiment. The polarization of the laser light, and hence the polarization of the emitted electrons, was changed for every filling-accelerating-extracting cycle of about 5 s. Maintaining the electron polarization during the acceleration process was a difficult task. Depolarizing resonances were compensated by carefully centering the beam in the quadrupoles, by applying harmonic corrections of resonance-driving magnetic fields, and by fast changes of the accelerator optics (tune jumping) at specific energies. After the slow extraction, the spin was rotated in a solenoid and two bending magnets into the longitudinal direction. At an electron energy of 2.335 GeV, a longitudinal electron polarization at the radiator target of 64% (51%) was achieved in the 2009 (2007) beam time.

**Circularly-polarized photons: ** The longitudinally-polarized electrons with energy $E_{0}$ impinged on a target and produced bremsstrahlung. The polarization of the electron beam was partly transferred to the photon beam yielding a photon polarization [80] of

6 $$\begin{aligned} P_{\odot }=\frac{4x-x^2}{4-4x+3x^2}P_\mathrm{e^-}\quad \text{ with } x=\frac{E_{\gamma }}{E_{0}}\,. \end{aligned}$$

The polarization had its maximum at the highest tagged-photon energies ($64\%$ at 2286 MeV for the 2009 beam time) and then decreased toward lower energies ($19\%$ at 600 MeV).

**The Møller Polarimeter: ** The Møller target was mounted inside of the vacuum tank of the goniometer. The polarimeter served two purposes: It produced the bremsstrahlung and acted as a device to measure the longitudinal polarization of the beam electrons at the radiator position by exploiting the spin dependence of the electron-electron scattering process. The Møller target consisted of a $20\,\mu$m vacoflux foil, an alloy of 49 % Fe, 49 % Co and 2 % V. It was oriented at an angle of $\pm 20^\circ$ relative to the photon beam and could be flipped from the $+ 20^\circ$ position to the $- 20^\circ$ position. A solenoidal magnetic field of 0.08 T saturated the magnetization of the alloy. An electron polarization of the Møller target of ($8.163 \pm 0.067$) % was reached [68, 81].

The Møller detector consisted of two lead-glass counters, which were placed in the forward direction above and below the plane where the bremsstrahlung electrons were measured. Electrons from Møller scattering were separated from bremsstrahlung electrons by asking for two coincident hits; accidental hits in both lead-glass counters were measured in parallel and subtracted. The foil was flipped frequently between its $\pm 20^\circ$ positions to eliminate potential contributions from transverse components of the beam polarization. The relative systematic uncertainty of the polarization measurement was determined to be 3.3 %. This included the systematic uncertainty of target foil polarization, the simulated effective asymmetry coefficient of the Møller detector setup as well as of the analysis-related systematic uncertainties such as background contributions in the Møller measurement.

###  Proton polarization

The Bonn frozen-spin target [82] used beads of butanol (C$_4$H$_9$OH) as the target material and provided polarized protons. The protons of the hydrogen atoms within the butanol—carbon and oxygen are spinless—were polarized using the method of dynamic nuclear polarization (DNP). In the DNP process, the polarization of the free electrons in the target material was achieved by doping the material with paramagnetic radicals, and then transferring the polarization to the protons using microwave irradiation. This was done in an external magnetic field of 2.5 T at a temperature of $\sim$300 mK. Depending on the microwave frequency, inducing hyperfine transitions, the spin could be aligned parallel or antiparallel to the magnetic field. For the measurement with the Crystal Barrel detector, the polarizing magnet needed to be removed. During the data-taking, the polarization was preserved by a very thin superconducting coil of 500 $\upmu$m in thickness which provided a magnetic field of 0.6 T. At the same time, the temperature of the target was lowered to less than 70 mK. This allowed continuous measurements of several days with relaxation times for the polarization of $\sim$ 500 h. During the measurements, a mean polarization of +65%/–71% (2007) and +70%/–74% (2009) was reached. Not considering the polarization direction, a mean polarization of 71% with a systematic uncertainty of 2% was reached for the whole beam time.

**Fig. 3:**
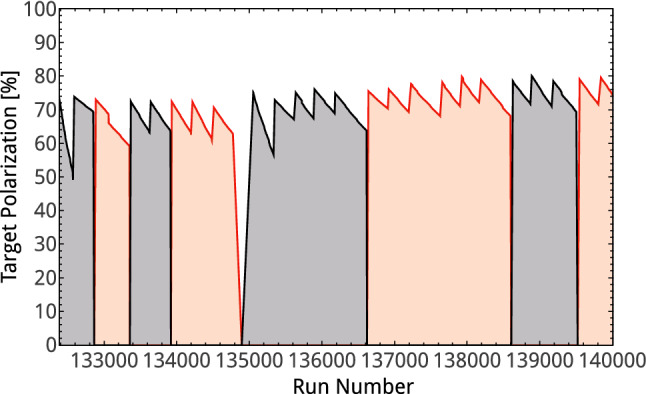
The target polarization for the two beam times (Sept. 2009 and Nov. 2009) with positive (red) and negative (black) polarization

The direction of the polarization was regularly changed (Fig. 3) either by flipping the direction of the external magnetic field or by adjusting the microwave frequency. Both methods were used to study systematic effects.

The target material could be replaced by the carbon foam target (see Sect. 3.4) inside the cryostat to determine the so-called dilution factor which determined the fraction of the polarizable protons in the butanol target for each energy and angular bin.

## Calibration and event reconstruction

For the final analysis, precise knowledge of the four-vectors of the different particles belonging to a certain event was needed. In a first step, the digital raw information stored in the analog-to-digital (ADCs) and time-to-digital converters (TDCs) of the detector systems had to be translated into energy, position and timing information of the final-state particles. In the following, the time and then the energy calibration of the different detector systems will be discussed before sections on particle reconstruction follow.

### Time Calibration

The time calibration ensured that hits occurring at the same time in the experiment were also reconstructed at the same event time (the time of flight for photons was set to zero for all detector systems). The time calibration proceeded in several steps. First, all values of the time-to-digital converters (TDCs) were transformed to relative times by a TDC-specific factor and the prompt peak in the TDC spectrum, defining the time at which the trigger occurred, was moved to $t=0$. In the Crystal Barrel/TAPS setup, the time provided by the tagger (96 scintillator bars) always defined the trigger time. Due to varying signal running times (cable lengths, electronics) of the different tagger channels, the trigger time was smeared out. Therefore, the above step provided only a first rough calibration.

**Fig. 4:**
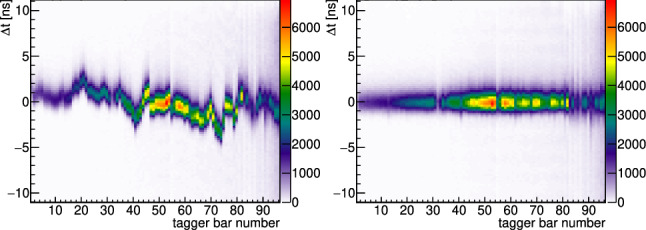
Time difference of the tagger bars relative to the Cherenkov detector before (left) and after (right) the second calibration step [83]

Time differences of various detector systems on the other hand are independent of the trigger time: $(t_1-t_\mathrm{trigger})-(t_2-t_\mathrm{trigger})=t_1-t_2$. Therefore, in a second step, the tagger bars and fibers were calibrated relative to the single-channel Cherenkov detector. The improvement of the tagger calibration is shown in Fig. 4.

The achievable time resolution was limited by the time-resolution of the Cherenkov detector (FWHM$\,\gtrsim \,$1 ns). Especially for the tagger bars and the BaF$_2$ crystals of the TAPS detector, a significantly better time resolution could be achieved. Therefore, they were cross-calibrated in an iterative procedure. Subsequently, all other detectors were calibrated using the calibrated tagger bars as a reference detector. In the last step, the time walk of the leading-edge discriminators of the Forward Plug (FP) was removed by an empirical energy-dependent correction function. In contrast to the FP, TAPS used constant-fraction discriminators, which made such a correction unnecessary.

**Fig. 5:**
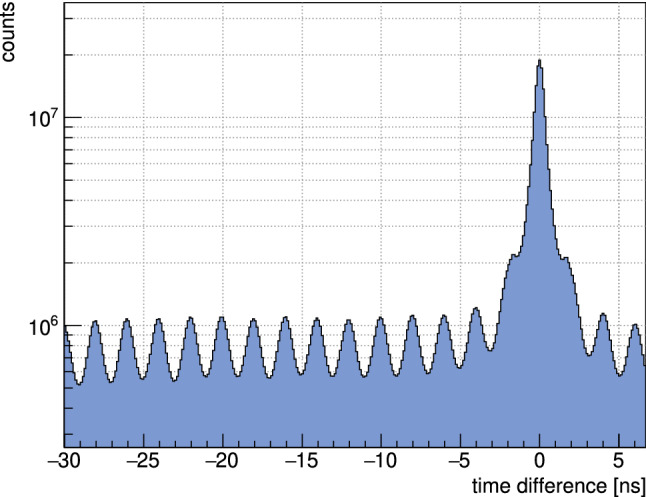
The time difference between two tagger bars after the calibration for all possible combinations. The periodic structure arises due to the 500 MHz bunch structure of the ELSA accelerator [83]

Measured relative to the tagger bars, the following resolution values were reached: FWHM = $0.635 \pm 0.003$ ns (tagger bars), $1.694\pm 0.06$ ns (tagger fibers), $0.872 \pm 0.006$ ns (TAPS BaF$_2$ crystals), $3.06\pm 0.05$ ns (TAPS plastic scintillators) $1.861 \pm 0.016$ ns (Forward Plug crystals), $4.434 \pm 0.013$ ns (plastic scintillators of the Forward Plug), and $2.093\pm 0.013$ ns (inner detector fibers) [83]. The quality of the final calibration can also be seen in Fig. 5, where the 500 MHz bunch structure of the ELSA accelerator is clearly observed.

### Energy calibration of the tagging system

During the regular data taking, the photon energy was calculated using the known ELSA electron beam energy, $E_0$, and the energy of the bremsstrahlung electron, $E_{e}$. The latter was determined by measuring the deflection angle of the electron in the magnetic field, $B_0$, using the scintillating bars and fibers of the tagging system (see Sect. 3.2). For the calibration, electrons with known beam energies $E^\mathrm{beam}_\mathrm{calib}$—smaller than during the regular data taking—were directly injected into the tagging system and their deflection angle was measured by the hit in a certain scintillator. By varying the magnetic field strength during the calibration measurements, the corresponding hit scintillator bars/fibers ($S_i$) were identified and using

$$\begin{aligned} \frac{E_{e}}{B_0}\,(S_i) \,=\, \frac{E^\mathrm{beam}_\mathrm{calib}}{B^\mathrm{calib}_i}\,(S_i)\,, \end{aligned}$$

the electron energy for a certain scintillator *i* at the nominal field $B_0$ during the data taking could be determined. The resulting *B*(*S*) correspondence was fit with a polynomial. Respective measurements were done for several different electron energies to study the systematics. The $E_\mathrm{e}(S)$ dependence was extended to lower $E_\mathrm{e}(S)$ via simulation for the area not reachable via the direct calibration procedure. The energy resolution of the tagging system ranged from $\varDelta E_\mathrm{e} \approx 0.5\,\% \,E_0$ at the highest electron (lowest photon) energies to $\varDelta E_\mathrm{e} \approx 0.1\,\% \,E_0$ at the lowest electron (highest photon) energies. The systematic uncertainty is of the order of half a fiber within the energy regime of the hardware calibration procedure, resulting in 4.2 MeV at 600 MeV and 0.9 MeV at 1750 MeV for an incoming electron beam energy of 2.335 GeV, for instance.

### Energy Calibration of the Calorimeters

**Crystal Barrel Calorimeter: ** 12-bit dual-range fastbus charge-to-digital converters (QDCs) were used to read out the Crystal Barrel calorimeter. To reach maximal resolution for low energetic photons, the signal was split with a ratio of 1:8 (high-range : low-range signal). This allowed the use of the whole QDC range for signals at low energies. At an energy of about 130 MeV for the Crystal Barrel and 260 MeV for the Forward Plug, the QDC switched from the low into the high range. To ensure a stable high-range/low-range factor and to determine this factor precisely, a light pulser was used. During dedicated light-pulser runs, pulsed signals of known relative intensity were sent via light guides to each of the crystal modules. The light guide emitted its light into the wave-length shifter behind the crystal, which was used to collect the light emitted by the crystal. These signals were recorded by the QDCs and were later used for the high/low-range calibration.

The Crystal Barrel shaper modules, placed in the signal path before the QDCs, allowed the adjustment of the pedestal via an offset current. The pedestals of all crystals were adjusted to one fixed value. Pedestal runs, which were performed at the start of each data-taking run, determined the individual pedestal value for each of the crystals. To suppress empty detector channels containing noise, only crystals with an ADC-entry 10 channels above this pedestal were written to tape during the data-taking. The pedestal value was subtracted from the measured ADC value as a first step in the analysis.

**Fig. 6:**
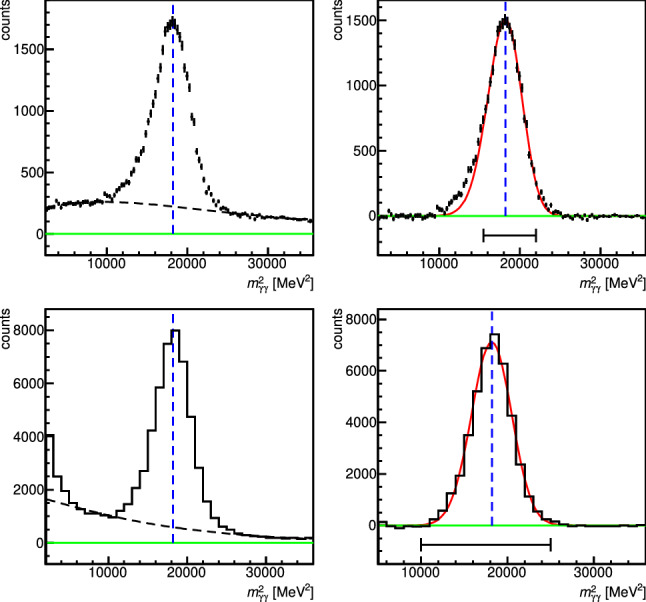
Examples of fits for selected crystals of the Crystal Barrel detector (top) and the TAPS detector (bottom). Each squared invariant mass is fitted with a Chebyshev polynomial of fifth order outside of the peak region to determine the background contribution (dashed line drawn over the entire mass range). The background function is then subtracted from the spectrum and the remaining peak is fitted by a Novosibirsk function [84] (red line, fit range indicated by black bar). For details on the calibration method, see [77, 85]

As a starting value for the calibration of each crystal, an overall low-range calibration factor of $c=0.033$ MeV per channel for the Crystal Barrel and of $c=0.061$ MeV per channel for the Forward Plug (or simply the values from the preceding beam time) were used. For the detailed calibration of the detector system, the well known $\pi ^0$ mass was used ($\pi ^0$ calibration), based on data selected as follows: All events with two neutral and not more than one charged hit in the detector system were initially selected using only events where the calorimeter hits were in the ADC low range. For an optimal energy and angle determination, only signals due to non-overlapping photon showers were used. The invariant mass of the two neutral hits showed a strong signal from the decay $\pi ^0\rightarrow \gamma \gamma$ (see Fig. 6). Within the calibration procedure, the squared invariant $\gamma \gamma$-mass was plotted separately for each crystal, which served as the central crystal 1. The spectrum was then fitted outside the peak region with a Chebyshev polynomial of fifth order to determine the background contribution. This background function was then subtracted from the spectrum and the remaining peak was fitted with a Novosibirsk function [84]. Based on the fitted mass a gain correction factor was determined for the selected central crystal. The procedure was repeated iteratively until the masses of the $\pi ^0$ signal in the spectra of all crystals were stable at the nominal pion mass (deviations of $\le$ 100 keV were tolerated). The high range was then calibrated by using the light-pulser system. Plotting the ADC channel versus the transmission (intensity) *T* of the light-pulser system allowed two straight line fits ($a_\mathrm{low}(T)$, $a_\mathrm{high}(T)$) and a conversion of the high range values into the low range system by

7 $$\begin{aligned} a_\mathrm{low} \,= & {} \, (a_\mathrm{high}\,-\,a_{\mathrm{high},\,T=0})\cdot g_{LP} \,+\, a_{\mathrm{low},\,T=0}\,,\nonumber \\ g_{LP} \,= & {} \, m_\mathrm{low}\,/\,m_\mathrm{high}\,, \end{aligned}$$

where $a_{\mathrm{low},\,T=0}~ (a_{\mathrm{high},\,T=0})$ and $m_\mathrm{low}~(m_\mathrm{high})$ denote the *y*-intercept and the linear slope of the low (high) range line, respectively.

**TAPS Calorimeter: ** Before each beam time, the TAPS detector was pre-calibrated to an energy deposit of 38 MeV using cosmic muons traversing 6 cm in a $\mathrm BaF_2$ crystal of the TAPS detector. The response of each TAPS crystal to the cosmic radiation did not only provide a first calibration factor for each crystal but also allowed a cross check of the constant fraction discriminator (CFD) threshold of the respective crystal. Only energy deposits passing the CFD threshold of the crystal were written to tape.

The positions of the pedestals for all crystals were determined regularly during the beam time since they showed some variations, e.g. with temperature. This was done repeatedly during the runs using a pulser-signal as trigger. Furthermore, based on the cosmic calibration and the pedestals determined for each data run, the TAPS data were also energy-calibrated using the $\pi ^0$-calibration method described above. Due to the limited angular coverage of the TAPS detector, this step was performed after the calibration of the Crystal Barrel calorimeter using events with one photon in TAPS and one photon in the Crystal Barrel calorimeter.

### Beam photon reconstruction

The incoming electrons, which produced bremsstrahlung on the radiator target, were deflected by the tagger dipole magnet. Their deflection angle was measured by the known position of scintillating bars and fibers (see Sect. 3.2), providing precise energy and time information for all tagged beam photons. Each electron hitting the tagger hodoscope left signals in up to three bars depending on the exact impact point of the electron. To reconstruct only one beam photon for each electron, all hits in neighboring bars, which occurred within a time interval of not more than $t_\text {diff} < 4$ ns, were combined into a single hit. Only hits affecting at least two scintillators, were retained to suppress background. Separately, the same was done for the scintillating fibers. Here, a missing fiber between two hits was allowed to account for inefficient fibers. Due to the worse time resolution, the time difference within a cluster of fibers was required to be below $t_\text {diff} < 7$ ns. For the high energetic electrons, the scintillating bars and fibers overlapped. In this region, a coincident hit of the scintillating bars and the fibers was required with a mean time difference between the two clusters of less than $t_\text {diff} < 4$ ns.

For the reconstructed beam photon, the mean time of the bar cluster or, in the overlap region, of bars and fibers, weighted by the respective time resolution, was used. The exact deflection angle was given by the mean position of the fibers or just by the scintillating bars, where no fibers were available. The energy of the beam photon was then determined by the tagging polynomial, as described in Sect. 5.2.

### Reconstruction of final-state photons

A photon, which interacted with the crystals of the calorimeters, created an electromagnetic shower which spread across the neighboring crystals. The Molière radius was 3.57 cm and 3.10 cm for the CsI(Tl) and BaF$_2$ crystals, respectively. The clusters of energy entries needed to be combined to finally determine the four-vector of the photon. A single-particle cluster was called a Particle Energy Deposit (PED).

**Crystal Barrel Calorimeter: ** In a first reconstruction step, the cluster algorithm was used to identify contiguous groups of crystals with energies above a single-crystal threshold of 1 MeV (well above noise level). In addition, a central crystal threshold of 20 MeV was used to ensure that only clusters above the FACE trigger-threshold of about 15 MeV were reconstructed. The direction of the incident particle (photon) can be reconstructed by an energy-weighted sum of the $\theta$ and $\phi$ coordinates of the crystals within the PED:

8 $$\begin{aligned} \theta _{PED}= & {} \frac{\sum _{i} w_i\theta _i}{\sum _i w_i}\,, \qquad \phi _{PED}=\frac{\sum _{i} w_i\phi _i}{\sum _i w_i}\,, \end{aligned}$$

9 $$\begin{aligned} w_i= & {} \max \left( 0; W_0 + \ln \frac{E_i}{\sum _{i} E_i} \right) \,, \end{aligned}$$

where the logarithmic weighting accounted for the exponentially-declining transversal shower profile. This resulted in a larger weight for the outer crystals and in an improved angular resolution. The cut-off value $W_0$ defined the lowest energy fraction for a crystal, which was used for the angle reconstruction. This value was optimized using Monte Carlo simulations ($W_0=4.25$). The four-momentum of a photon was then determined by assuming that it originated from the target center, and moved in the $\theta ,\,\phi$-direction of the electromagnetic shower.

So far only clusters produced by a single particle, resulting in a single-PED cluster, were discussed. However, energy deposits of different particles could also overlap, still producing a single connected cluster of crystals with energy deposits above the single-crystal threshold. Therefore, all clusters were scanned for local maxima above 20 MeV. If more than one maximum was found in a cluster, the cluster was considered a multi-PED cluster. Here, the 20 MeV threshold was used to reduce split-offs, which are clusters originating from a single particle, but with separate local maxima in the deposited energy due to shower fluctuations.

For the angle reconstruction in these overlapping clusters, only the crystal with the local maximum in energy and its direct neighbors were used in Eqs. (8, 9). Furthermore, the crystal energies $E_i$ were corrected with the calculated energy overlap expected. Here, the contribution of the second PED, calculated in terms of the distance of the crystal to the shower center of the second PED in units of the Molière radius, $d_{i\leftrightarrow \text {max}}/R_M$, was subtracted:

10 $$\begin{aligned} E_i^\text {corr} = E_i - E_\text {max}\cdot e^{-d_{i\leftrightarrow \text {max}}/R_M}\,. \end{aligned}$$

The ratio of the PED central energies determined how the total cluster energy was distributed among the two overlapping PEDs. Multi-PED clusters with more than two local maxima were very rare and neglected in the analysis.

In addition, a correction function for the PEDs was used: The segmentation of the detector, important for the angular resolution, as well as other support structures of the detector introduced also dead material into the detector. Part of the energy of the shower was therefore not measured. This loss depended on the energy as well as on the direction of the particle. To correct for these losses an energy correction function was determined from simulations and was applied to each PED.

**TAPS Calorimeter: ** The reconstruction of the TAPS calorimeter was very similar to the one of the Crystal Barrel calorimeter, with three distinct differences: (1) TAPS provided not only an energy but also a time signal for each crystal. In the reconstruction, all crystals that were considered belonging to the same cluster were required to fulfill $t_\mathrm {diff} < 5$ ns. The TAPS detector was located in the forward direction and for this reason, its crystals detected much more low-energetic background from the beam halo. (2) To prevent background events from reaching the read-out chain, the thresholds of the TAPS constant-fraction discriminators were set higher than the thresholds for the Crystal Barrel calorimeter. The two inner rings, which were closest to the primary photon beam, had thresholds of 17 MeV, while the outer-ring thresholds were set to 13 MeV. In addition, the central crystal of a cluster was required to have an energy of more than 20 MeV, and the total energy of a cluster had to be above 25 MeV. (3) The crystal axes of the TAPS detector were not oriented toward the target direction. Hence, an additional position- and energy-dependent correction factor was necessary. These were part of the energy-correction function developed for TAPS, again based on Monte-Carlo simulations.

### Identification of charged particles

Three different detectors served to identify a charged particle: (1) The inner scintillating-fiber detector covering polar angles of $167^\circ> \theta > 21^\circ$, (2) the plastic scintillators in front of the forward detector for angles $27.5^\circ> \theta > 11.2^\circ$, and (3) the plastic scintillators in front of the TAPS crystals, closing the polar-angle gap down to $1^\circ$.

**Inner scintillating fiber Detector: ** The two inner layers were rotated with respect to the beam axis (see Sect. 3.5) while the outer layer had straight fibers (parallel to the beamline). Signals in two of the three layers were hence sufficient to define a hit and its position. First, the hits were clustered layer-wise. Between two fibers, a single missing fiber was allowed; The time difference of the signals could not exceed 14 ns. The hits in the respective layers were then combined to two- or three-layer hits if the time differences were again within 14 ns. The time and position of the hits were given by the mean time and the mean position of all involved fibers. The polar and azimuthal angle of the resulting crossing point was then calculated and the four-vector determined under the assumption that the particle originated from the target center.

A cluster in the Crystal Barrel calorimeter was identified as a charged-particle cluster if the angle between the trajectory from the target center to the cluster and of the trajectory from the target center to the inner detector hit was less than $12^\circ$ for $|\varDelta \phi |$ and for $|\varDelta \theta |$. If a calorimeter time was available in the area of overlap between the inner scintillating fiber detector and the Forward Plug, an additional time cut of $\varDelta t \le 15$ ns was applied.

**Plastic Scintillators of the Forward Plug: ** Two layers of plastic scintillators were placed in front of the forward part of the Crystal Barrel detector. Each plastic scintillator covered $\varDelta \phi =12^\circ ,\,\varDelta \theta =6^\circ$. The second layer was turned by $6^\circ$ with respect to the first layer and doubled the angular resolution to $\varDelta \phi =6^\circ$. Hits in both layers of scintillators within 20 ns were required to define a charged particle. The center of the overlapping scintillators was used to determine the polar and azimuthal angle. The particles’ four-vectors were calculated assuming that they originated from the target center.

A forward-detector hit was marked as a charged particle if the azimuthal angle difference between the trajectory from the target center to the cluster and to the scintillator hit was less than 14$^{\circ }$, the polar-angle difference was less than 10$^{\circ }$, and the time difference between the hits in both layers was less than 20 ns.

**Plastic scintillators of the TAPS Detector: ** A plastic scintillator was also located in front of each crystal of the TAPS detector. Hits in these scintillators were clustered based on the time and the location of the detector. However, since the plastic scintillators did not overlap, hits in multiple neighboring detectors were rather rare.

Since the TAPS detector was a planar detector, its crystals were not oriented toward the target center, and trajectories, first penetrating a neighboring scintillating plate, were possible. Therefore, a particle in the calorimeter was identified as a charged particle if the distance between the two trajectories of the calorimeter cluster and the reconstructed scintillator at the surface of TAPS was less than 6.5 cm and $|\varDelta t| \le 15$ ns.

###  Event selection

The reaction $\gamma p\rightarrow p\pi ^0\rightarrow p\gamma \gamma$ was reconstructed by a series of kinematic cuts. Events with exactly two neutral and one charged particle were used. The charged particle, always considered to be a proton, was required in order to suppress background from reactions off neutrons. Two classes of events were considered in the analysis, 3-PED events as well as 2-PED events with a charged particle identified in the inner detector. In the case of 3-PED events, all events with three clusters in the calorimeters were considered for further analysis. Exactly one of them had to be identified as a charged particle (see Sect. 5.6). To include also events with low-energy protons, which did not produce a cluster in the Crystal Barrel calorimeter above the energy threshold of 20 MeV, two uncharged PEDs in the calorimeters and a hit in the inner detector were considered as well. In the subsequent analysis, the proton was treated as a missing particle for both the 2-PED and the 3-PED events. Its four-momentum was calculated from the known $\gamma p$ initial state and from the final-state photons measured in the calorimeters.

In a next step, a $\pm 2\sigma$ cut on the invariant $\gamma \gamma$ mass around the nominal $\pi ^0$ mass, and on the missing mass around the nominal proton mass was applied. Since the width of the missing mass distribution increased with energy, the cuts were adjusted for each incident photon energy bin. Furthermore, the azimuthal angle between the the proton and the $\pi ^0$, was required to be 180$^{\circ }$ within an energy dependent $\pm 2\sigma$ window (coplanarity). To remove untagged events originating from photons below the tagging threshold (due to random coincidences), the beam photon energy was calculated from the kinematics of the reaction using the four-momentum of the $\pi ^0$, the proton mass, and the condition that the total transverse momentum in the event was zero. This value for the photon energy was then compared to the measured photon energy in the tagging system. A cut on the calculated photon energy of $E_\gamma >550$ MeV was applied, which was 50 MeV below the first investigated photon energy bin.

A time coincidence between the tagger hit and the reaction products was required. A subtraction of the random time-background was performed using side-band subtraction to remove time-accidentals due to the high rate in the tagging system.

**Fig. 7:**
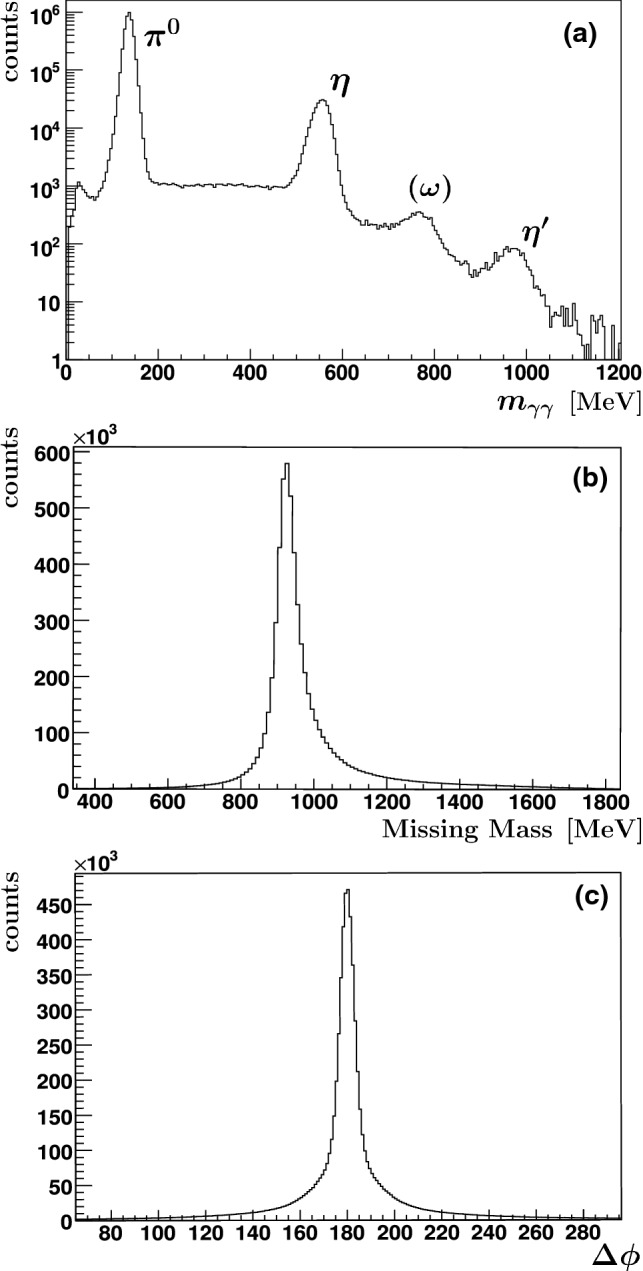
Invariant $\gamma \gamma$ mass (**a**), missing mass (**b**) and coplanarity (**c**) spectrum. All cuts except for the one on the respective spectrum have been applied and random coincidences have been subtracted. The $\omega$, even though decaying into $\pi ^0\gamma \rightarrow 3\gamma$, is visible in the invariant $\gamma \gamma$ mass spectrum; here, either one low-energetic $\gamma$ remained undetected or two photons merged into one cluster accidentally interpreted as one photon

The resulting distributions for the butanol data are shown in Fig. 7 after applying all cuts with the exception of those for the respective distributions. Figure 7a shows the invariant $\gamma \gamma$ mass distribution (log-scale). A clear peak with little background is visible for the $\pi ^0$. It is followed by peaks for the $\eta \rightarrow \gamma \gamma$, $\omega \rightarrow \pi ^0\gamma \rightarrow 3\gamma$ (with one undetected low-energetic $\gamma$ or two merging photons in one cluster accidentally interpreted as one photon), and $\eta ^\prime \rightarrow \gamma \gamma$. The missing-mass distribution (Fig. 7b) shows a clear peak at the proton mass.

Background is visible not only above but also below the proton mass peak, although accidental time-background has been subtracted. The latter is not present for data taken with a liquid hydrogen target since the missing mass X in a reaction $\gamma p \rightarrow \pi ^0 X$ can only be larger but not smaller than the mass of the proton. The low missing-mass events are due to the Fermi motion of the nucleons bound in C or O. A similar effect is visible in the coplanarity spectrum (Fig.7c), where the peak observed at 180$^\circ$ has a significantly larger width than expected for a hydrogen target, two Gaussian distributions of different widths overlap. These observations became important when the fraction of events produced off bound nucleons in the data set was determined (see Fig. 10).

**Data sample: ** After all cuts, about $1.2\cdot 10^6$ ($4.6\cdot 10^6$) events due to the reaction $\gamma p\rightarrow \pi ^0 p$ from the butanol target were retained in the 2007 (2009) beam time. Additionally, $0.2\cdot 10^6$ events taken with the carbon foam target survived after applying all cuts.

## Data analysis and results

**Fig. 8:**
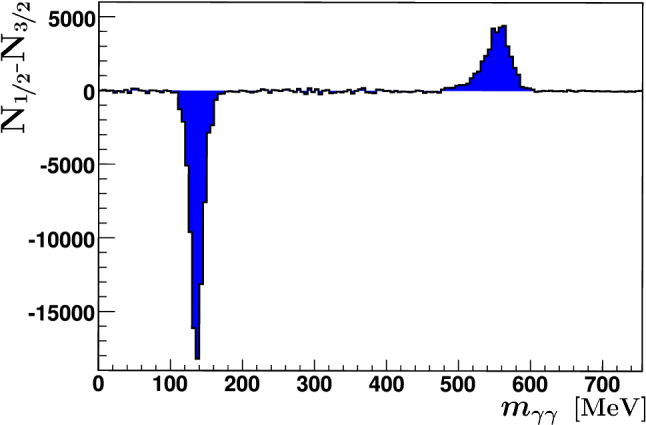
$N_{1/2}-N_{3/2}$ for the invariant mass spectrum. All events with energies above $E_\gamma =550$ MeV are included

**The count rates: ** The double-polarization observable *E* was determined via formula (2). Figure 8 shows the count rate difference $N_{1/2}-N_{3/2}$ of the invariant $\gamma \gamma$ mass spectrum integrated over all energies and angles. A pronounced dip is visible at the pion mass and a peak at the $\eta$ mass. In the photoproduction of neutral pions, the $\sigma _{3/2}$ cross section obviously exceeds the $\sigma _{1/2}$ cross section in the investigated energy range, while the reverse holds true in the photoproduction of $\eta$ mesons. This fact illustrates the importance of investigating different reaction channels which highlight different resonance contributions.

**Fig. 9:**
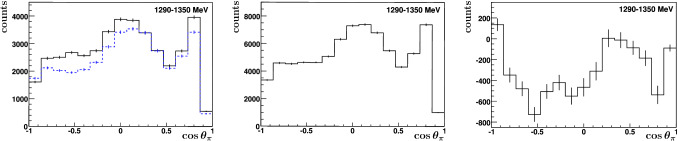
Left: $N_{1/2}$ (dashed blue) and $N_{3/2}$ (solid black) separately; Middle: $N_{1/2}+N_{3/2}$; And right: $N_{1/2}-N_{3/2}$ as function of $\cos {\theta _\pi }$

The observable *E* is a function of the photon energy and of $\cos \theta$, where $\theta$ denotes the angle of the $\pi ^0$ relative to the photon beam axis in the center-of-mass frame. Figure 9 shows the count rates $N_{1/2}$ and $N_{3/2}$, the difference $N_{1/2}-N_{3/2}$, and the sum $N_{1/2}+N_{3/2}$ for one specific energy bin, $E_\gamma = 1290 - 1350$ MeV.

For $\gamma p \rightarrow p\pi ^0$, photoproduction off unpolarized carbon and oxygen nuclei does not depend on the helicity of the photon beam. The contribution of the carbon and oxygen nuclei to the count rate difference $N_{1/2}-N_{3/2}$ vanishes. Yet, the carbon and oxygen nuclei do contribute to the sum $N_{1/2}+N_{3/2}$. Naively, one might expect that all protons bound in the carbon and oxygen nuclei (32 out of the 74 nucleons in C$_4$H$_9$OH) contribute to the sum $N_{1/2}+N_{3/2}$. This is, however, not true. The kinematics of the reaction off bound nucleons differs from the reaction of free protons due to the Fermi motion, and the contribution of the carbon and oxygen nuclei after all selection criteria are applied is smaller than naively expected. For this reason, the *dilution factor* needs to be determined for each bin in photon energy and $\cos \theta$. It depends significantly on the cuts applied in the data selection.

**The dilution factor: ** The dilution factor, *d*, for the reaction of interest is defined as the ratio of the number of events produced off hydrogen nuclei ($N_\mathrm{H}$, free protons) to the number of events produced off all nucleons in the butanol target ($N_\mathrm{C_4H_9OH}$) in the final data set:

11 $$\begin{aligned} d(E_{\gamma },\theta )=\frac{N_\mathrm{H}(E_{\gamma },\theta )}{N_\mathrm{C_4H_9OH}(E_{\gamma },\theta )}\,. \end{aligned}$$

The number of events produced off hydrogen is given by the number of events produced off butanol minus the number of events which are assigned to the production off the nucleons bound in carbon and oxygen ($N^{But}_\mathrm{C}$). The latter is the number of $\pi ^0p$ events which were produced off bound C/O-nucleons and which passed all cuts of the analysis chain:

12 $$\begin{aligned} N_H(E_{\gamma },\theta )=N_\mathrm{C_4H_9OH}(E_{\gamma },\theta )-s(E_{\gamma })\cdot N_\mathrm{C}(E_{\gamma },\theta ),\nonumber \\ \end{aligned}$$

where $s(E_{\gamma })$ is a scaling factor, which was determined using the additional data taken with the carbon foam target ($N^{But}_\mathrm{C} = s\cdot N_\mathrm{C}$). Events produced off hydrogen and carbon/oxygen have different kinematic distributions. This allowed us to separate the two reactions by performing additional measurements using a carbon foam target within the polarized target cryostat filled with helium. This assumes that the difference between photoproduction off carbon and off oxygen does not play a significant role. This is supported by the study in Ref. [86], which showed that events due to pion production off carbon and oxygen nuclei have similar kinematic distributions.

**Fig. 10:**
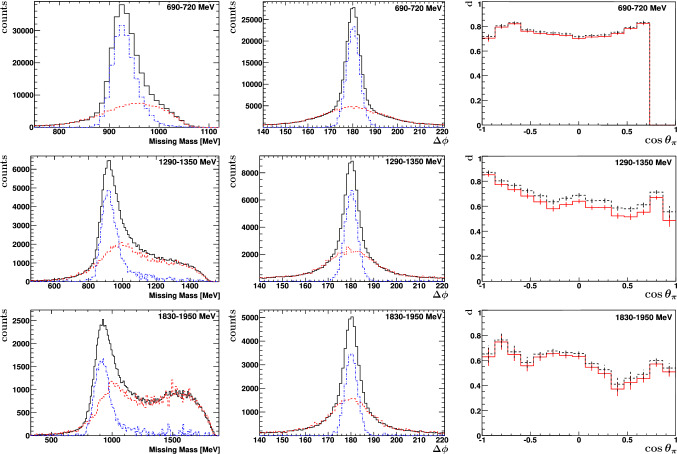
Missing-mass spectra (first column), coplanarity spectrum (second column) for $E_{\gamma } = 690-720$ MeV (1. row), $E_{\gamma }=1290-1350$ MeV (2. row), and $E_{\gamma }=1830-1950$ MeV (3. row). Black solid: butanol data, red dashed: scaled carbon data, blue dashed-dotted: $N_B-sN_C$ data. Last column: Dilution factor for both methods: missing mass in black dashed and coplanarity in red solid. Using the missing mass method results in a slightly larger dilution factor. This difference might be explained due to slightly different conditions in the butanol and carbon measurement (e.g. target positioning), which have a larger impact on the missing mass compared to the coplanarity spectrum. The difference in the dilution factor is considered in the systematic uncertainty

The scaling factor $s(E_{\gamma })$ was determined using two different methods. The first column of Fig. 10 shows missing-mass spectra for three ranges of photon energy integrated over the solid angle: The solid histogram (black) represents the missing-mass distribution from the butanol target (MM$_{\,\mathrm C_4H_9OH}$), and the dashed (red) histogram represents the missing mass distribution from carbon (MM$_{\,\mathrm C}$) already scaled to the distribution from butanol. To determine the scaling factor $s(E_{\gamma })$, the distribution from carbon was fitted to the background events at the left side of the missing-mass spectrum (see Fig. 10). Subtracting the scaled distribution measured with carbon from the distribution measured with butanol results in the dashed-dotted (blue) histogram.

In the second method, the coplanarity spectrum is fitted in a similar way. Due to Fermi motion, the coplanarity spectrum does not only show a Gaussian peak of small width at 180$^\circ$ as expected for hydrogen, but also an additional rather broad distribution. The carbon distribution, following this broad additional distribution, was fitted to the coplanarity spectrum for $100^\circ \le \varDelta \phi \le 160^\circ$ and $200^\circ \le \varDelta \phi \le 260^\circ$ and the respective scaling factor was again determined. Subtracting the scaled carbon from the butanol distribution results in the dashed-dotted (blue) histogram with hardly any visible background.

After determining the scaling factors for the different energy bins, the dilution factor could be derived as a function of photon energy and $\cos \theta$ using Eqs. (11) and (12). The dilution factor is shown as a dashed black histogram in the third column of Fig. 10 for the first (MM) and as solid red histogram for the second (coplanarity) method. It is nearly 0.8 at the lowest photon energy and decreases to 0.5 above 2 GeV.

The double-polarization observable *E* was finally determined using the dilution factor from the second method (coplanarity spectra). Compared to the missing-mass distribution, the number of butanol and carbon events in the fit region are larger and the coplanarity spectrum does hardly change its shape with energy in contrast to the missing-mass spectrum. Differences in the dilution factor from the two methods were taken into account as a systematic uncertainty.

**The polarization: ** The last step to be considered in the determination of *E* is the polarization of the circularly-polarized photon beam and the longitudinally-polarized target (Eq. 2). Each event in the count-rate difference $N_{1/2}-N_{3/2}$ has been weighted by $\frac{1}{P_TP_\odot }$. The target polarization $P_T$ has been determined from NMR measurements before and after each polarization phase and interpolated in between the two points using the measured relaxation time. Thus, the polarization of the target is known at each point in time.

The electron beam polarization was determined using the Møller polarimeter (see Sect. 4.1). The polarization transferred from the electron to the photon is a function of its energy and was determined using Eq. (6). It is denoted as $P_\odot$.

**Fig. 11:**
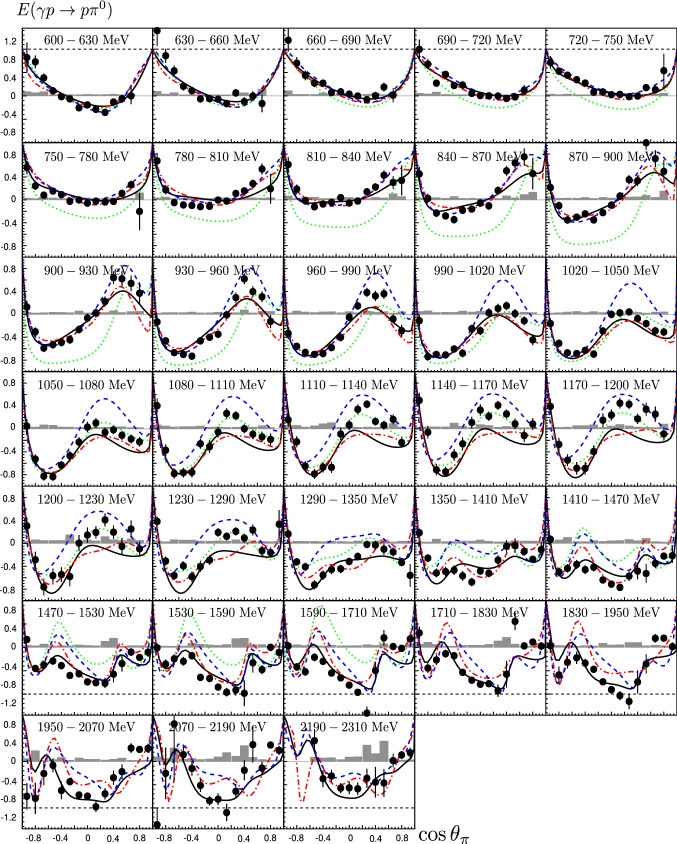
Double-polarization observable E for different beam energies from 600 to 2310 MeV. The data are compared with the PWA predictions BnGa 2011-02 (black solid line) [24], JüBo 2015-B [26] (blue dashed line, 2015-B included already the *G*-data bins given in [47]), MAID 2007 (green dotted line) [29], and the SAID prediction for solution CM12 (red dashed-dotted line) [25]. The predictions are shown for the center energy of the bin. The gray area represents the systematic uncertainty

**Fig. 12:**
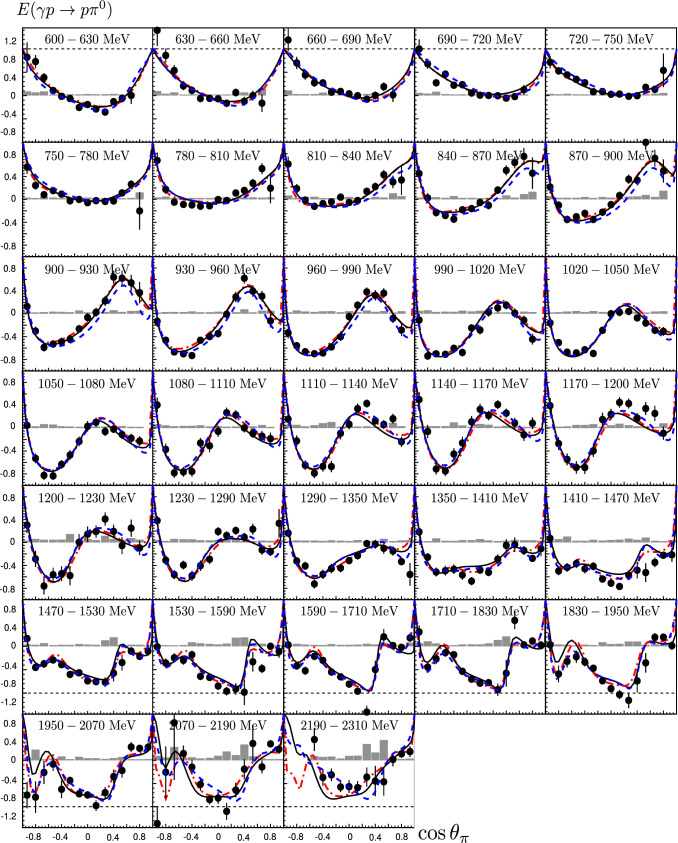
Double-polarization observable E for different beam energies from 600 to 2310 MeV. The data are compared to the PWA results from three fits including this data set: BnGa 2014-02 (black solid line) [67, 87], JüBo 2016-1 (blue dashed line) [67], and SAID 2015 (PD03) (red dashed-dotted line) [67]. The PWA-curves are shown for the center energy of the bin. The gray area represents the systematic uncertainty

**The double-polarization observable** *E*: The results on *E* using Eq. (2) are presented in Figs. 11 and 12 in comparison to different partial wave analysis solutions (predictions (Fig. 11) and fits (Fig. 12)). For $E_\gamma <1230$ MeV, the data are shown in 30-MeV-wide bins. For $1290<E_\gamma <1590$ MeV, 60-MeV-wide bins are used, three 120-MeV-wide bins cover the energy range up to 2310 MeV. The solid-angle coverage is arranged in 15 bins in $\cos \theta$. The total statistical uncertainty includes contributions from the statistical uncertainties in the event numbers $N_{1/2}$ and $N_{3/2}$ and contributions from the statistical uncertainty in the determination of the dilution factor. The systematic uncertainties, shown as gray bands in Figs. 11 and 12, were obtained from a comparison of the results from different analyses: Two different methods to determine the dilution factor were used as discussed above and different cut values were applied in the data selection. The systematic uncertainty in the target and beam polarization was estimated to be 2 % and 3.3 %, respectively. The different contributions to the systematic error were added in quadrature.

Angular momentum conservation guarantees that

$\sigma _{3/2}\,(\cos \theta =\pm 1)$ vanishes at forward and backward angles, hence $E\,(\cos \theta =\pm 1)=1$. This requirement is compatible with the experimental findings. As a function of energy, the $\cos \theta$ distributions change steadily. At low photon energies (<900 MeV), we expect only a few low-spin resonances to contribute, and hence no oscillatory behavior, in agreement with the experimental findings. At higher photon energies, more structure in the angular distribution is expected and seen in the data.

**Partial wave analyses methods: ** Different partial wave analyses have been performed by the SAID, MAID, Jülich-Bonn (JüBo) and Bonn-Gatchina (BnGa) groups which can be compared to the data. We outline shortly the basic ingredients used in the fits. A more detailed description of the partial wave analysis methods of the four PWA groups and references to the data used in the fits are presented in [67].

SAID (GWU/INS) [28] uses a Chew-Mandelstam formulation of the scattering matrix, which is parametrized in the form of a K-matrix [23]. A fit to data on $\pi N$ elastic scattering, charge exchange and $\pi ^-p\rightarrow \eta n$ determines a *hadronic rescattering matrix* which encodes the hadronic channel coupling (or the effects of rescattering in the final state), and returns masses, widths, and $\pi N$ branching ratios of 12 $N^*$ and 9 $\varDelta ^*$ resonances. The model was extended to include data on photoproduction (CM12) [25] and the photocouplings of seven low-mass $N^*$ and 5 $\varDelta ^*$ resonances were determined.

The MAID group [29] combines the 13 four-star resonances with masses below 2 GeV known in 2006 [88] and a common background in a unitary formalism to fit data on pion photo- and electroproduction from Bates/MIT, ELSA/Bonn, MAMI/Mainz, and Jefferson Lab. Masses, widths and $\pi N$ branching ratios of the resonances are taken from [88].

A dynamical coupled-channel approach is employed by the Jülich-Bonn (JüBo) group. The model guarantees unitarity and analyticity, and incorporates general S-matrix principles. In a first step, data on $\pi N$ elastic scattering and on $\pi N\rightarrow \eta N, K\varLambda ,$ and $K\varSigma$ were fitted [89]; these data define the poles and residues of the 24 $N^*$ and $\varDelta ^*$ resonances. In a second step, data on pion photoproduction were used to determine the photocouplings of the contributing resonances [27].

The Bonn-Gatchina group [24] employs covariant amplitudes in a K-matrix formalism to perform combined analyses of most known data on single and double-meson production in photon- and pion-induced reactions; weak-decay modes (like $\gamma N$ in production and decay) are treated in the form of production (*P*) or decay (*D*) vectors which do not contribute to rescattering. Two equivalent classes of solutions compatible with all data included at that time were presented in [90].

As discussed below, the data presented here have been included together with further new polarization data into the different partial wave analyses. The respective results are summarized below. A detailed discussion on the PWA-results is given in [67].

**Partial wave analyses—predictions: ** In Fig. 11 the results are compared to the predictions of different partial wave analyses performed by the SAID (CM12), MAID, Jülich-Bonn (2015-B) and Bonn-Gatchina (BnGa 2011-02) groups. The comparison shows a wide spread of the predicted results and thus, underlines the need for new polarization data. In the low-energy region (600–780 MeV), only the BnGa, JüBo and SAID predictions are compatible with the new data. In describing the cross section, MAID underestimates the contributions with helicity $A_{1/2}$ and overestimates those with helicity $A_{3/2}$. In the 800–950 MeV range, the JüBo, BnGa and SAID predictions agree fairly well with the data. From 1.0 to 1.4 GeV, the general trend of the data is predicted by all PWAs, even though quantitatively, the agreement is fair, at most. Above this energy, most PWA predictions (except BnGa) deteriorate further. While all predictions show three local minima, the JüBo, SAID and MAID predictions have a pronounced maximum at $\cos \theta \approx -0.45$. Likely, it originates from the interference of $\varDelta (1950)7/2^+$ production and background amplitudes; it is too strong in these three analyses since the contributions of other resonances and their interference with $\varDelta (1950)7/2^+$ are not included in the predictions. In the MAID online version [91], the enhancement in the prediction disappears when the $N(1520)3/2^-$ helicity couplings are reduced by a factor of 4. The qualitative agreement between the data and the BnGa prediction is surprisingly good in the high-mass region.

**Partial wave analyses—new fits:** Figure 12 shows the data on *E* again but now with new fits by the PWA groups. The fits take into account not only the new data on *E* presented here, but also other recently published polarization data: new data on $\gamma p\rightarrow \pi ^0p$ have been reported from Mainz, a measurement of the polarization transfer from a polarized photon beam to a recoiling nucleon, $C_x$, [54] and low-energy data on $d\sigma /d\varOmega$ and $\varSigma$ [39]; the beam asymmetry $\varSigma$ has been reported from JLab data on $\gamma p\rightarrow \pi ^0p$ and $\gamma p\rightarrow \pi ^+n$ [40]; the variables $P,C_x,C_z$ have been measured for one energy relevant here, $E_\gamma =1845$ MeV, at JLab [44]; data on *G* [48] and on *T*, *P*, *H* [41, 42] have been taken at Bonn.

**Fig. 13:**
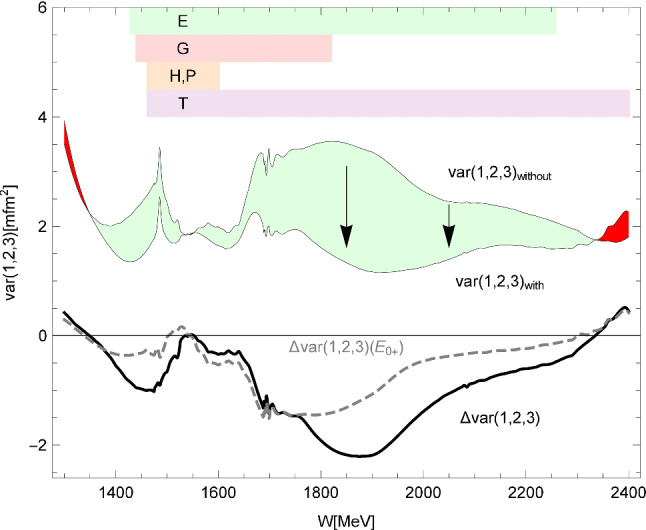
The variance of all the three PWAs (JüBo, SAID, BnGa) summed over all $\gamma p\rightarrow \pi ^0 p$ multipoles up to $L=4$. The range covered by the new double-polarization observables are indicated by shaded areas. Over the largest part of the energy range, the new data have enforced an improvement of the overall consistency. The improvement is displayed as light green area and, separately as difference of the variance. The contribution to the improvement from the $E_{0+}$ wave is shown as the dashed curve. Ranges with an overall deterioration are marked in red (figure taken from [67]). The spike slightly below $W=1.5$ GeV reflects discrepancies in the description of the $\eta p$ threshold effect in the different PWAs. A wider peak below $W=1.7$ GeV might stem from slightly different $N(1680)5/2^+$ properties used in the three PWAs. Both become less pronounced when the new data are included in the fits

**Fig. 14:**
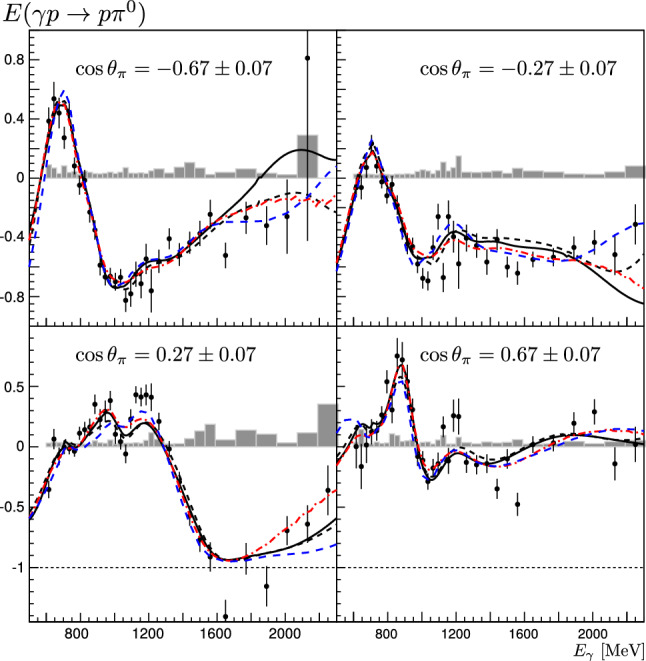
The energy dependence of E for four different angles, compared to different solutions of the different PWAs: BnGa2014-02 (black solid), BnGa2014-01 (black dashed) [67, 87], JüBo 2016-1 (blue dashed) [67], SAID 2015 (PD03) (red dashed-dotted) [67]. All solutions shown are based on fits, which included the data presented here. The gray area represents the systematic uncertainty.

All four PWAs are capable of reproducing the data quantitatively, but small differences remain. A detailed comparison of the PWA results of the different PWA groups based on the the data presented here as well as on the data mentioned above is given in [67]. Here we only summarize the main result. In the limit of a complete database with a complete angular coverage and large statistics, choosing the same overall phase, the amplitudes of the different PWAs should converge to the same physical solution. Since the new data represent a significant improvement of the existing database and represent an important step toward a complete experiment, one would expect a convergence of the solutions of the different PWAs toward the same solution even though the amplitudes do not yet reach the point of being really identical. Figure 13, taken from [67] compares the different amplitudes in terms of multipole amplitudes (for details see [67]). For this purpose, the variance between two models, 1 and 2, has been calculated as the sum over the squared differences of the 16 (complex) $\gamma p\rightarrow \pi ^0 p$ multipoles ${{{\mathcal {M}}}}$ up to $L=4$:

13 $$\begin{aligned} \mathrm {var}(1,2) = \frac{1}{2} \sum _{i=1}^{16}({{{\mathcal {M}}}}_1(i) - {{{\mathcal {M}}}}_2(i))({{{\mathcal {M}}}}_1^*(i) - {{{\mathcal {M}}}}_2^*(i))\,.\nonumber \\ \end{aligned}$$

**Fig. 15:**
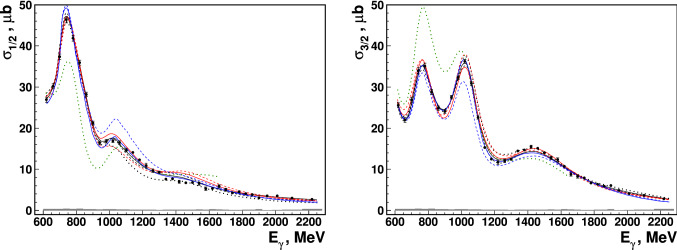
$\sigma _{1/2}$ and $\sigma _{3/2}$ and PWA results from different solutions including the data presented here: BnGa 2014-02 (black solid) [67, 87], 2014-01 (black dashed), SAID (PD03) [67] (red solid, curve calculated used solution PD03 for *E* and CM12 for the cross section), JüBo 2016-01 [67] (blue solid), and not including this data: BnGa 2011-02 (black dotted), SAID CM12 (red dashed), JüBo 2013-01 (blue dashed), MAID 2007 (green dotted). The gray area represents the systematic uncertainty

**Fig. 16:**
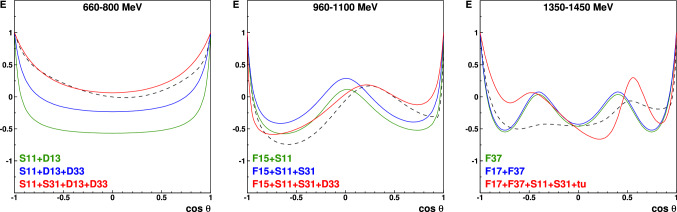
Black dashed: BnGa fit to the data (BG2014-02 [67, 87]). Different colors: single partial wave contributions within the description of the BnGa-PWA as indicated in the figures. For further details, see text

The mean of the variance

$$\begin{aligned} \mathrm {var}(1,2,3)=\frac{1}{3}\cdot \displaystyle \sum _{i,j=1,i\ne j}^{3}\mathrm {var}(i,j) \end{aligned}$$

of the three PWAs (JüBo, SAID and BnGa), before and after the new data are included in the fit, is shown in Fig. 13. The overall spread of the three partial wave analyses is reduced considerably due to the impact of the new polarization observables of Refs. [42, 48] and this work. As visible in Fig. 13, a significant fraction of the improvement stems from the $E_{0+}$ multipole exciting the $J^P=1/2^-$ wave (and thus the resonances *N*(1535), $\varDelta (1620)$, *N*(1650), *N*(1895), and $\varDelta (1900)$).

Figure 14 displays the energy dependence of the double polarisation observable *E* for four different angular bins in comparison to the final solutions of the different PWAs. At high photon energies, significant differences between the different PWAs are visible. Obviously additional data, extending the energy range of the polarization observables discussed here, improving their statistics, and the measurement of additional polarization observables are needed to further constrain the PWA amplitudes to one common solution.

**Spin-dependent cross sections:** From the double-polarization observable *E*, the spin-dependent total cross sections $\sigma _{1/2}$ and $\sigma _{3/2}$ can be calculated via

14 $$\begin{aligned} \sigma _{1/2}= & {} \sigma _0(1+E)\,,\end{aligned}$$

15 $$\begin{aligned} \sigma _{3/2}= & {} \sigma _0(1-E)\,. \end{aligned}$$

For the unpolarized cross section $\sigma _0$, the BnGa2014-02 fit to the data was used. The calculation of the total cross section requires the integration over the full solid angle. In the angular range, where no data points exist, the double-polarization observable *E* was extrapolated to the unmeasured angular range using the BnGa fit. The resulting total cross sections for $\sigma _{1/2}$ and $\sigma _{3/2}$ are shown in Fig. 15, in comparison to the different PWA predictions.

The two distributions are very different. In the second resonance region, covering the photon energy range from 660 to 900 MeV, the $\sigma _{1/2}$ peak is significantly stronger than the peak in $\sigma _{3/2}$. This is due to the sizable contribution of the $N(1535)1/2^-$ resonance which contributes to $\sigma _{1/2}$ only. Some early partial wave analyses, MAID and SAID-SN11, underestimated the $N(1535)1/2^-$ contribution to $\gamma p\rightarrow N\pi$, and assigned this intensity to the $N(1520)3/2^-$ resonance. The third resonance region covers the energy range from 900 to 1200 MeV. In this region, the MAID and SAID-SN11 and, to a smaller extent, the SAID-CM12 and BnGa 2011-02 partial wave analyses overestimate the $\sigma _{3/2}$ contribution, at the expense of $\sigma _{1/2}$, while JüBo underestimated the $\sigma _{3/2}$ contribution.

The description of the data by the most recent fits of the different PWA groups (which included the data presented here) improved quite significantly. The fourth resonance region, 1200 $<E_\gamma<$ 1700 MeV, is dominated by $\sigma _{3/2}$ but none of the fits is fully satisfactory. Likely, the statistical significance of the high-mass data in Fig. 12 is not large enough to constrain the multi-channel fit [67].

**The dominant partial wave contributions:** It may be illuminating to compare the results on *E* with simple models. In Fig. 16, we display the result of the BnGa fit with the predictions based on a few partial waves. The observed angular distribution of *E* for 660 $<E_\gamma<$ 800 MeV exhibits a single minimum almost reaching $E\approx 0$ just above $\cos \theta =0$. If there was only a contribution from $J^P=1/2^-$ ($S_{11}$), $E=1$ would hold for all values of $\cos \theta$. This is obviously not observed in the data. Adding the amplitude for $N(1520)3/2^-$ ($D_{13}$) (with the helicity couplings as determined in the fit) leads to a much deeper minimum than observed. It requires contributions from $\varDelta (1620)1/2^-$ ($S_{31}$) and $\varDelta (1700)3/2^-$ ($D_{33}$) to arrive close to the expected curve. The remaining asymmetry requires additional contributions from odd waves, e.g. from $\varDelta (1600)3/2^+$ ($P_{33}$).

In the 960 $<E_\gamma<$ 1100 MeV range, the angular distribution of *E* is characterized by two minima which originate from the interference of $J^P=1/2^-$ ($N(1535)1/2^-$, $\varDelta (1620)1/2^-$) and $J^P=5/2^+$ ($F_{15}$) ($N(1680)5/2^+$). The forward-backward asymmetry due to the interference of even and odd waves becomes more pronounced adding $\varDelta (1700)3/2^-$.

The photon energy interval from 1350 to 1450 MeV exhibits an angular distribution of *E* with three local minima, or two intermediate maxima. Such a pattern can be reproduced by the $J^P=7/2^+$ partial wave ($N(1990)7/2^+$ ($F_{17}$), $\varDelta (1950)7/2^+$ ($F_{37}$)). However in the data, the local minimum at $\cos \theta \approx -0.45$ is considerably lower than the minimum at $\cos \theta \approx 0.9$. Adding $J^P=1/2^-$ waves ($N(1535)1/2^-$, $\varDelta (1620)1/2^-$) and *t*- and *u*-channel contributions, the data represented by the final BnGa curve (dashed black) are still not yet reasonably well reproduced: many partial waves are required (and do contribute in the BnGa fit).

## Summary

We have reported a measurement of 467 data points on the helicity asymmetry $E=(\sigma _{1/2}-\sigma _{3/2})/(\sigma _{1/2}+\sigma _{3/2})$. The data cover the $E_\gamma$ energy range from 600 to 1230 MeV in 30 MeV wide bins, 1230–1590 MeV in 60 MeV wide bins, and the range from 1590 to 2310 MeV in 120 MeV wide bins. The bin widths are chosen in view of the available statistics and of the dependence of the angular distributions on the photon energy. The data are presented in 15 slices in $\cos \theta$; in most energy bins, the solid angle coverage is almost complete.

The data presented here, and other new polarization data measured at Bonn, JLab, and Mainz have been included in the PWAs of the BnGa, JüBo, and SAID groups. The new data have had a significant impact on the fit. While the previous predictions exhibit large discrepancies among themselves and with the data, there is good agreement between data and all new fits. Smaller discrepancies will need further studies, both experimentally and in new partial wave analyses.

## Data Availability

This manuscript has associated data in a data repository [Authors’ comment: The data are made available via HEPData.]

## Footnotes

1 The crystal with the highest energy deposit in the cluster.
